# RESIDENT RIGHTS: BALANCING SAFETY AND AUTONOMY IN LONG-TERM CARE

**DOI:** 10.1093/geroni/igad104.0985

**Published:** 2023-12-21

**Authors:** Angela Perone

**Affiliations:** University of California, Berkeley, Berkeley, California, United States

## Abstract

Long-term care regulations require staff to keep residents safe while also respecting their autonomy. Building on street-level bureaucracy theory (Lipsky, 2010), which focuses on how front-line workers use discretion to implement ambiguous policies, this study examines how staff at various levels (floor staff, mid-managers, top management) balance these rights. This study employs a multi-method qualitative extended comparative case study of three levels of staff in two long-term care facilities (independent, corporate structure). Data includes in-depth semi-structured staff interviews (n=80), participant observation (n=8 months), and review of facility policy documents (n=376) and legal data (i.e., laws and regulations). Autonomy and safety presented conflicting rights for staff in three situations: fall prevention, food intake/refusal, and medication management. Staff exercised discretion via interpersonal conversations, documentation, and organizational lenses/resources and used interprofessional team approaches across staff hierarchies, especially among floor staff and mid-managers. Even when upper-managers sought external guidance (e.g., attorneys/risk managers), they deferred to the discretion of floor staff and mid-managers to communicate and implement decisions. Team approaches across staff hierarchy were less common when conflicting rights arose in medication management. Differences in organizational structure became relevant only when conflicting rights regarding autonomy and safety arose for food intake. This presentation will discuss the study’s strategies for employing coding teams and data software to analyze large datasets across diverse methodological approaches. It will also discuss ethical dilemmas with sharing large qualitative datasets as secondary data of populations deemed “vulnerable” by IRB.

